# The Current Status and Future Potential of Theranostics to Diagnose and Treat Childhood Cancer

**DOI:** 10.3389/fonc.2020.578286

**Published:** 2020-11-19

**Authors:** Alex J. Poot, Marnix G. E. H. Lam, Max M. van Noesel

**Affiliations:** ^1^ Department of Radiology and Nuclear Medicine, University Medical Center Utrecht, Utrecht, Netherlands; ^2^ Department of Solid Tumors, Princess Maxima Center for Pediatric Oncology, Utrecht, Netherlands

**Keywords:** theranostics, childhood cancer, radiopharmaceuticals, nuclear imaging (e.g. PET, SPECT), nuclear therapy

## Abstract

In theranostics (i.e., therapy and diagnostics) radiopharmaceuticals are used for both therapeutic and diagnostic purposes by targeting one specific tumor receptor. Biologically relevant compounds, e.g., receptor ligands or drugs, are labeled with radionuclides to form radiopharmaceuticals. The possible applications are multifold: visualization of biological processes or tumor biology *in vivo*, diagnosis and tumor staging, therapy planning, and treatment of specific tumors. Theranostics research is multidisciplinary and allows for the rapid translation of potential tumor targets from preclinical research to “first-in-man” clinical studies. In the last decade, the use of theranostics has seen an unprecedented value for adult cancer patients. Several radiopharmaceuticals are routinely used in clinical practice (e.g., [^68^Ga/^177^Lu]DOTATATE), and dozens are under (pre)clinical development. In contrast to these successes in adult oncology, theranostics have scarcely been developed to diagnose and treat pediatric cancers. To date, [^123/131^I]meta-iodobenzylguanidine ([^123/131^I]mIBG) is the only available and approved theranostic in pediatric oncology. mIBG targets the norepinephrine transporter, expressed by neuroblastoma tumors. For most pediatric tumors, including neuroblastoma, there is a clear need for novel and improved radiopharmaceuticals for imaging and therapy. The strategy of theranostics for pediatric oncology can be divided in (1) the improvement of existing theranostics, (2) the translation of theranostics developed in adult oncology for pediatric purposes, and (3) the development of novel theranostics for pediatric tumor-specific targets. Here, we describe the recent advances in theranostics development in pediatric oncology and shed a light on how this methodology can affect diagnosis and provide additional treatment options for these patients.

## Introduction

**Figure 1:**
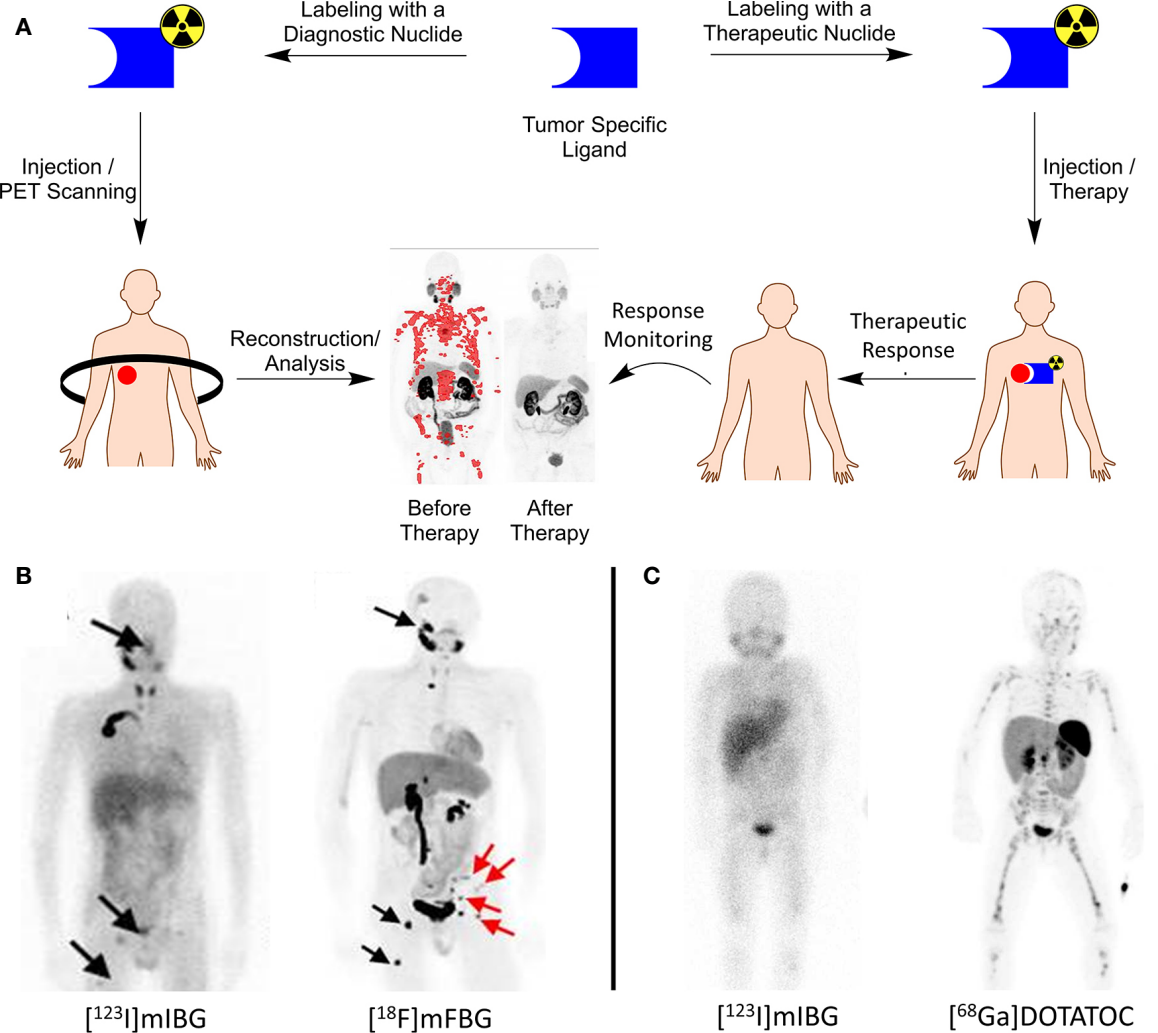
**(A)** Theranostics concept explained. A tumor-specific ligand can be used for both imaging and therapy, dependent on the nuclide of choice. PET images before/after therapy in a prostate cancer patient diagnosed and treated with [^68^Ga/^177^Lu]PSMA. PET image adapted from SNMMI image of the year 2018 by Hofman et al.; **(B)** left, [^123^I]mIBG SPECT image of a neuroblastoma patient with lesions indicated with black arrows; right, [^18^F]mFBG PET image of the same patient showing greater contrast and additional lesions that were not observed with [^123^I]mIBG. Image adapted from Pandit-Taskar et al. (1); **(C)** left, [^123^I]mIBG SPECT image of a neuroblastoma patient with only vague tumor uptake; right, [^68^Ga]DOTATOC PET image of the same patient showing SSTR-2A expression, greater contrast and additional lesions. Patient is treated with [^177^Lu]DOTATATE with an additional survival of 24 months (unpublished data, UMC Utrecht).

Theranostics in nuclear medicine includes the use and application of two identical or very closely related radiopharmaceuticals for *ther*apy and diag*nos*is. In oncology, tumor-specific substrates, receptor ligands, or drugs can serve as lead for theranostic development when labeled with specific radionuclides for imaging or therapy (**Figure 1A**). As the molecular structure of both the diagnostic and therapeutic radiopharmaceuticals are identical, diagnostic images can become predictive for therapeutic response because the biological characteristics and binding potential of both are similar, irrespective of the radionuclide (2–4).

For diagnosis, positron emission tomography (PET) is a nuclear imaging technique that enables the visualization and quantification of molecules equipped with positron emitting radionuclides. The most used radionuclide for imaging is Fluorine-18 (^18^F) in the form of [^18^F]FDG. [^18^F]FDG PET visualizes increased carbohydrate uptake in tissue, e.g., tumor tissue, and is important for diagnosis, staging and treatment monitoring. For PET tracer development, any molecule that displays tumor-specific targeting can be used, including small molecules, peptides or biologicals. Radionuclides used for PET tracer development are, among others, Carbon-11 (^11^C) and Fluorine-18 (^18^F) facilitating small molecule labeling, Gallium-68 (^68^Ga) for peptide radiolabeling, and Copper-64 (^64^Cu) or Zirconium-89 (^89^Zr) for the labeling of monoclonal antibodies (mAbs) and other biologicals. PET imaging enables studying the distribution and kinetics of labeled molecules and the biochemical and physiological processes. Molecular imaging by means of PET can, thus, facilitate and guide cancer treatment in many ways (5, 6). Currently, PET is the most sensitive technique for nuclear imaging; it requires nanomolar amounts of the radiopharmaceutical for imaging. These nanomolar amounts will not induce pharmacological effects, hold minimal risks for toxicity, and are described as the micro-dosing concept. Micro-dosing allows for fast translation of novel PET tracers into clinical trials in small “first-in-man” or phase 0 studies, when produced under good manufacturing practice (GMP). Single photon emission computed tomography (SPECT) is an alternative nuclear imaging technique and enables the visualization of γ-emitting radionuclides and was the basis for early theranostics development, where, among others, the different radionuclides of iodine were used for imaging (e.g., Iodine-123 (^123^I) and Iodine-131 (^131^I)).

Therapeutic radiopharmaceuticals for treatment of cancer are predominantly labeled with β-emitting radionuclides. The radionuclide ^131^I, Lutetium-177 (^177^Lu) and Yttrium-90 (^90^Y) are frequently used for this purpose. The emitted β-particles travel 1–12 mm through tissue upon decay while losing energy and causing cytotoxic damage to the cell to induce apoptosis. Alternatively and more recently, α-emitting radionuclides, e.g., Astatine-211 (^211^At) or Actinium-225 (^225^Ac) were explored for therapy (7–9). The high energy deposition and a limited range of the α-particles in tissue (0.005–0.11 mm) result in very strong cytotoxic and therapeutic effects. Nowadays, α-emitting radionuclides become more widely available, research toward the development of therapeutic radiopharmaceuticals with these radionuclides is emerging, and first-in-man studies are expected in the near future.

Successful theranostics have been developed for somatostatin receptor positive neuroendocrine tumors with [^68^Ga/^177^Lu]DOTATATE and prostate-specific membrane antigen (PSMA) positive prostate cancer patients as prime examples (10–13). Currently, for childhood cancers and more specifically norepinephrine transporter (NET) positive neuroblastoma tumors, [^123/131^I]meta-iodobenzylguanidine ([^123/131^I]mIBG) is the only available theranostic to date (14–16). Despite the proven value of theranostics in adult oncology, its potential was minimally explored for childhood cancer and is still at its infant stage. However, many opportunities and applications present themselves. In this review, we discuss different strategies for theranostics development for childhood cancer and divided these into (1) the existing theranostics and improvement thereof, (2) theranostics developed for adult oncology and translation thereof for childhood cancer, and (3) the development of novel theranostics for specific pediatric tumor targets. By describing the recent advances in theranostics research we discuss how it can affect diagnosis and therapy for childhood cancer in the future.

## Current Theranostics in Pediatric Oncology

[^123/131^I]mIBG is the only theranostic currently available for routine clinical use to image and treat neuroblastoma tumors that express the norepinephrine transporter (NET). mIBG is a structural analog of the neurotransmitter norepinephrine and is actively transported into the tumor by NET. Inside the cell, mIBG is stored in the cytoplasm, mitochondria, and in vesicular monoamine transporter (VMAT)-coated and neurosecretory vesicles (17–21). [^123^I]mIBG SPECT imaging is currently the standard of care to diagnose primary tumors and distant metastases in neuroblastoma and for staging and disease response evaluation after treatment. In total, approximately 95% of neuroblastoma tumors are [^123^I]MIBG avid. The remaining 5% of tumors are either well-differentiated ganglioblastoma or very undifferentiated neuroblastoma with little or no NET transporter expression. Although [^123^I]mIBG SPECT has a high specificity and sensitivity, it also has disadvantages being poor image resolution, long scanning times, and iodine-driven thyroid toxicity. Accompanied by imaging, [^131^I]mIBG initially showed therapeutic effectiveness in bulky tumors (22). Subsequently, it was shown that [^131^I]mIBG was feasible and effective in the first treatment of high-risk neuroblastoma patients (23). However, two systematic reviews failed to show a survival advantage for [^131^I]mIBG treated patients (24, 25). In two studies [^131^I]mIBG was combined with busulfan and melphalan followed by autologous stem cell rescue. For both, acceptable toxicity in highly pretreated patients and encouraging responses were observed. This has led to the implementation of this combination for ultra-high-risk patients who failed to respond adequately during induction treatment for high-risk neuroblastoma. The current European SIOPEN VERITAS study explores the role of [^131^I]mIBG in combination with topotecan and stem cell rescue followed by another high-dose consolidation with Buslfan and Melphalan and a second stem cell rescue. The aim is to increase the survival of these ultra-high-risk patients. In conclusion, [^131^I]mIBG treatment is still under investigation and its definitive role has not been determined. In addition to the discussion on therapeutic response, patients receiving [^131^I]mIBG also suffer from iodine uptake in the thyroid and increased risk for long-term thyroid dysfunction or secundary thyroid cancer. Last, after [^131^I]mIBG administration, patients need to live in isolation for 5–7 days and strict precepts for 2–3 weeks. Despite the value of [^123/131^I]mIBG as a theranostic, both imaging and therapy have serious disadvantages and limitations that steer the research toward novel approaches.

An ^18^F-labeled analog of [^123^I]mIBG, [^18^F]meta-fluorobenzylguanidine ([^18^F]mFBG]) has long been proposed as a possible PET alternative for the imaging of NET-positive neuroblastoma tumors (26). The radionuclide ^18^F is a cyclotron produced β^+^-emitter with a short range *in vivo*, resulting in a high image quality. Furthermore, PET-CT (or PET-MRI) images can be analyzed quantitatively for tracer distribution. ^18^F-labeled radiopharmaceuticals are, therefore, ideal for high-resolution diagnosis, faster acquisition, and low radiation burden. Until recently, however, the production of [^18^F]mFBG has been challenging. It requires a nucleophilic aromatic substitution of an electron-rich molecule (27, 28). Recent advances and novel radiofluorination reactions now give access to the production and clinical translation of [^18^F]mFBG (1, 29).

Pandit-Taskar et al. reported the first clinical results with [^18^F]mFBG, described a biodistribution and dosimetry study in neuroblastoma patients, and compared the results with [^123^I]mIBG. In all five neuroblastoma patients, [^18^F]mFBG scored better than [^123^I]mIBG with respect to lesion counts, improved image quality, and the absence of any thyroid uptake (**Figure 1B**). These encouraging results gave rise to additional and more extensive clinical testing of [^18^F]mFBG as an alternative to [^123^I]mIBG as the current gold standard (**Table 1**) (30, 31).

**Table 1:** Theranostics under preclinical development and in clinical trials for pediatric cancers.

Molecular Target	Pediatric Cancer	Theranostic	Development Phase	Pediatric Clinical Trial Number	Refs
Norepinephrine	Neuroblastoma	[^123/131^I]mIBG,	Routine Care	Multiple Trials	(14–16, 22–25, 78),
Transporter		[^18^F]mFBG,	Phase I/II	NCT02348749	(29, 30)
		[^211^At]mABG	Preclinical		(34)	
Somatostatin	Neuroblastoma	[^68^Ga]DOTATATE,	Phase I/II	NCT04040088	(39–42),
Receptor 2A		[^177^Lu]DOTATATE,	Phase I/II		
		[^68^Ga]OPS202,	Phase I/II		
		[^177^Lu]OPS201,	Phase I/II		
C-X-C Chemokine	Neuroblastoma	[^68^Ga]Pentixafor,	Early Phase I		(48, 50),
Receptor 4	Rhabdomyosarcoma	[^177^Lu]Pentixather	Early Phase I		
	Glioblastoma				
	ALL &AML				
Fibroblast	Glioblastoma	[^68^Ga]FAPI,	Phase I/II		(56, 57),
Activation Protein		[^177^Lu]FAPI	Preclinical		
Ganglioside D2	Neuroblastoma	[^89^Zr]Dinutuximab,	Preclinical		(68–70)
	Osteosarcoma	[^68^Ga]WHWRLPS	Preclinical		
	Glioblastoma				
B7-H3 (CD276)	Pontine Glioma	[^124/131^I]8H9	Phase I/II	NCT03275402/	(75, 76),
	Neuroblastoma	(omburtamab)		NCT01502917/	
				NCT04022213	

In addition to improved imaging, research is now focused on the development of an improved alternative for [^131^I]mIBG therapy. ^131^I is a β^–^-emitter with a t_1/2_ of 8.04 days. Furthermore, when [^131^I]mIBG is metabolized and ^131^I is released, it will accumulate in the thyroid. Therefore, the thyroid is blocked as a preventive action by administration of excess iodine to avoid undesired effects. As an alternative for ^131^I, ^211^At has been explored. ^211^At is an α-emitter with a t_1/2_ of 7.2 h and a range of 0.005–0.11 mm in tissue. These physical properties cause very strong cytotoxic and therapeutic effects. Furthermore, ^211^At does not accumulate in the thyroid and potentially will not cause any undesired damage (32, 33), As ^211^At has benefits over ^131^I, [^211^At]meta-astatobenzylguanidine ([^211^At]mABG) was reported as an alternative for [^131^I]mIBG for the treatment of NET positive tumors. To date, [^211^At]mABG has only been evaluated in preclinical models on PC12 xenografted mice (**Table 1**). [^211^At]mABG showed a dose-dependent tumor regression and increased survival compared to the control animals. It should, however, be noted that a high dose of [^211^At]mABG caused the death of the animals. Therefore, the toxicity profile and maximum tolerated dose of [^211^At]mABG needs to be assessed and compared to [^131^I]mIBG. An important additional note is the availability of ^211^At to produce [^211^At]mABG, which may become a practical concern. ^211^At can only be produced by high-energy cyclotrons, of which a few are installed worldwide, and thereby the access is limited (34).

## From Adult Oncology to Pediatric Oncology

Theranostics available in routine clinical care are a rich source of potential theranostic candidates in pediatric oncology.

The somatostatin receptor (SSTR) family is one of the first discovered and most successful targets identified for which theranostics were developed. To date, 5 subtypes of SSTR (i.e., SSTR-1, 2A, 3, 4, and 5) are characterized. In particular, SSTR-2A is important with high expression levels for neuroendocrine tumors. It is involved in secretion, proliferation, and the induction of apoptosis (35). For pediatric cancers, SSTR-2A expression was reported for neuroblastoma tumors by Alexander et al. as well as for neuro-oncological malignancies (e.g., glioblastomas and medulloblastomas) (36–38). Analogs of somatostatin, the natural ligand of SSTR-2A, have successfully been developed to inhibit neuroendocrine tumor growth. Radiolabeling of these compounds led to the development of [^68^Ga]DOTATATE as a PET tracer and received FDA approval in 2016 (**Table 1**). In 2018, [^177^Lu]DOTATATE (Lutathera, AAA/Novartis) was approved as a therapeutic agent to treat SSTR-2A positive tumors. As DOTATATE is an SSTR-2A agonist, it stimulates the receptors, potentially causing undesired tumor growth. To circumvent these agonistic effects, the theranostics pair [^68^Ga]OPS202/[^177^Lu]OPS201 (Ipsen) was developed as an SSTR-2A antagonist and is currently in Phase I/II trials (**Table 1**) (10, 11, 39, 40). Because SSTR-2A expression was also validated for neuroblastomas and neuro-oncological malignancies and with several theranostics available, a straightforward translation to pediatric oncology is feasible. Small-scale experimental pilot studies were reported for these pediatric cancers with [^68^Ga]DOTATATE, and results are encouraging (41). This warrants further clinical studies on imaging and treating SSTR-2A positive pediatric cancers with these theranostics in the near future (**Figure 1C**) (42).

Another theranostic candidate target that was extensively explored in adult oncology is the C-X-C chemokine receptor 4 (CXCR4). The expression levels of CXCR4 and its natural ligand, CXCL12, are correlated to tumor development and metastasis and were validated for breast cancer, prostate cancer, lung cancer, colorectal cancer, and primary brain tumors (43). By immunohistochemical staining, CXCR4 expression was also demonstrated for neuroblastomas, rhabdomyosarcomas, glioblastomas, and hematological malignancies (44–47). To date, several CXCR4-targeting drugs are under (pre)clinical development, e.g., Ulocuplumab, PRX177561, AMD3100, and Plerixafor, which demonstrates that CXCR4 targeting is clinically feasible and relevant. For theranostic development, the PET tracer and cyclic-pentapeptide [^68^Ga]Pentixafor (Scintomics) is currently the most advanced and under investigation in multiple Phase I clinical trials (**Table 1**) (48). Labeling of pentixafor with ^177^Lu or ^90^Y to obtain the therapeutic counterpart of the diagnostic led to a strongly decreased affinity for the target receptor. This affinity could be restored after small molecular adaptations to the pentixafor scaffold and resulted in the successful development of [^177^Lu]Pentixather (**Table 1**) (43, 49, 50). [^68^Ga]Pentixafor and [^177^Lu]Pentixather are candidates for clinical trials in pediatric patients as well as CXCR4 is reported for these tumors.

A target that recently received much attention is the fibroblast activation protein α (FAP) (51). FAP is a serine protease that is selectively expressed in the stromal fibroblasts of the tumor, which is often observed for breast cancer, colon cancer, and pancreatic cancers (52). FAP expression is observed in glioblastomas and can be a valuable theranostic target for pediatric cancers. FAP-specific inhibitors (FAPI) have been developed based on quinoline scaffolds. For diagnostic purposes, promising results were obtain after radiolabeling with ^68^Ga (53, 54). In particular, [^68^Ga]FAPI-04, -21, and -46 resulted in high-contrast images, and as a proof-of-concept, 28 different tumor types were visualized with [^68^Ga]FAPI-04 (55, 56). All FAPI compounds allow radiolabeling with ^177^Lu too to obtain the corresponding therapeutic radiopharmaceutical. Preclinical studies with [^177^Lu]FAPI-21 and -46 in tumor-bearing mice gave promising results (**Table 1**) (57). As FAP is also expressed by glioblastomas, these theranostics have potential for the diagnosis and treatment of pediatric cancers.

Monoclonal antibodies (mAbs) and mAb-fragments had unprecedented impact on the treatment of cancer patients. However, clinical benefit is usually only achieved in a percentage of the patient population. The application of ^89^Zr-labeled mAbs as ImmunoPET tracers has become increasingly important to visualize these compounds *in vivo* and assess the distribution, kinetics, and the biochemical and physiological behaviour (4, 58). Nowadays, more than 75 clinical trials are ongoing with ^89^Zr-labeled mAbs and the radiolabeling can be achieved *via* generic methods (59, 60). Despite the clinical impact of ImmunoPET with [^89^Zr]mAbs for adult oncology and other indications, ImmunoPET with available [^89^Zr]mAbs has barely been explored for pediatric cancers. The only reported application of ImmunoPET was [^89^Zr]bevacizumab in diffuse intrinsic pontine glioma to study vascular endothelial growth factor (VEGF) excretion and the potential to treat these patients with bevacizumab (61). Though ImmunoPET in pediatric cancer patients is not common, it should be anticipated that this methodology can also have an impact for these patients in the future.

## Specific Theranostic Targets in Pediatric Oncology

Pediatric cancers have a distinct biological profile with unique molecular targets that are not expressed in adult cancers. These targets embody unique opportunities for the diagnosis and treatment of pediatric cancers, but due to small patient populations, it remains a challenge to identify them and develop theranostics against these targets.

A target of interest for theranostic development is ganglioside D2 (GD2). GD2 is a glycosphingolipid and selective cellular marker that is expressed by neuroblastomas, osteosarcomas, and glioblastomas (62, 63). Though its exact function is still not fully understood, it is assumed that it plays a crucial role in cell adhesion, migration, and tumor metastasis. Dinutuximab (Unituxin^®^, United Therapeutics) is FDA approved, and Dinutuximab beta (Qarziba^®^, EUSA Pharma) is EMA approved for the treatment of GD2-positive neuroblastoma tumors (64, 65). Despite increased survival rates from 46% to 66%, for high-risk neuroblastoma patients, 30% of the patients will relapse independent of the GD2 expression levels (**Table 1**) (66). Several radiopharmaceuticals have been developed to image GD2-positive tumors. ^64^Cu-labeled hu14.18K322A showed clear accumulation and retention in preclinical osteosarcoma models, and [^89^Zr]dinutuximab was mentioned as a PET tracer in meeting abstracts (67–69). In addition to radiolabeled mAbs, Müller et al. reported on the development of [^68^Ga]DOTA- WHWRLPS heptapeptide and demonstrated its accumulation in neuroblastoma xenografted mice (70). Though encouraging, clinical translation of these radiopharmaceuticals has yet to be achieved.

More recently, B7-H3 (CD276) has become a validated pediatric cancer target for immunotherapy in pontine gliomas and neuroblastomas (71, 72). To date, two mAbs were developed, Enoblituzumab (MacroGenics) and Omburtamab (Y-mAbs), to treat B7-H3 positive tumors (73, 74) Based on these immunotherapeutics, attempts at the development of theranostics are reported. Especially with 8H9 (i.e., Omburtamab) multiple clinical trials are ongoing. The theranostics pair [^124/131^I]8H9 is investigated for B7-H3 positive pontine glioma tumors and a modest survival benefit was reported (**Table 1**) (75, 76). As specific brain tumors (e.g., gliomas) express B7-H3, it is important that passage and delivery of the radiopharmaceutical across the blood–brain barrier is achieved. As such, radiolabeled [^124/131^I]Omburtamab is ideal to investigate drug targeting in these patients. As B7-H3 is acknowledged as a pan-tumor target, theranostics targeting B7-H3 might become of general importance for childhood cancer.

## Considerations and Requirements for Nuclear Medicine in Childhood Cancer

The application of theranostics for the diagnosis and treatment of childhood cancers is in its infancy. With the availability of radiopharmaceuticals and theranostics for the various adult cancers, there is a lot of potential to translate and directly apply these for childhood cancers. Clinically available SPECT/PET tracers and therapeutic radiopharmaceuticals can directly be applied for pediatric cancers when the target is present and validated for the respective tumor type. Examples include SSTR-2A, CXCR4, and FAP-positive tumors. As childhood cancers have unique target expression profiles, with GD2 and B7-H3 as examples, novel theranostics can be developed for these yet unexplored targets. Unique target-finding programs are in place to unveil novel childhood cancer-specific biological features for which theranostics can be developed. A critical note and challenge is that target expression of cancers in general cannot always be directly correlated to positive imaging and treatment results. Preclinical research programs are, therefore, required to validate target expression and the potential of the target against which to developed theranostics.

A successfully developed theranostic that shows potential in preclinical studies warrants clinical translation. To achieve that, the theranostic needs to be produced under GMP to guarantee product quality and patient safety (77). Clinical translation of developed theranostics is relatively straightforward as procedures and GMP production facilities are widely available.

## Conclusion

Theranostics have unprecedented value to diagnose and treat cancers. Many novel theranostics are under development and expected to enter clinical trials and care in the near future. For the diagnosis and treatment of childhood cancers, theranostics research is still in its infancy, but following the path of adult oncology, its value is promising. They are expected to become additional and valuable tools to diagnose and treat childhood cancers.

## Author Contributions

AP drafted the manuscript. All authors contributed to the article and approved the submitted version.

## Funding

Sponsored by UMC Utrecht and Princess Máxima Center for Pediatric Oncology, Netherlands.

## Conflict of Interest

The authors declare that the research was conducted in the absence of any commercial or financial relationships that could be construed as a potential conflict of interest.
